# Disseminated Intravascular Coagulation With a Predominant Thrombotic Phenotype in Severe Plasmodium falciparum Malaria: A Case Report

**DOI:** 10.7759/cureus.104046

**Published:** 2026-02-22

**Authors:** Eugeniu Gisca, Miguel G Santos, Simone Costa, Catia R Santos, Ana Araújo

**Affiliations:** 1 Critical Care Medicine, Unidade Local de Saúde da Região de Leiria - Hospital de Santo André, Leiria, PRT

**Keywords:** atrial thrombosis, dic in malaria, disseminated intravascular coagulation (dic), plasmodium falcifarum, severe malaria

## Abstract

Severe *Plasmodium falciparum* malaria may be complicated by disseminated intravascular coagulation (DIC) with heterogeneous clinical manifestations. We report the case of a 61-year-old man with recent travel to Angola who presented with severe *Plasmodium falciparum* malaria complicated by DIC with a predominant thrombotic phenotype. On admission, he exhibited profound thrombocytopenia, markedly elevated D-dimer levels, metabolic acidosis, shock, and evidence of multiorgan dysfunction. Peripheral blood smear demonstrated parasitemia exceeding 25%.

Despite prompt initiation of intravenous artesunate, anticoagulation, and comprehensive organ support in the intensive care unit, the patient developed progressive peripheral ischemia requiring right above-knee amputation. Although parasitemia rapidly declined and initial haemodynamic stabilisation was achieved, his hospital course was further complicated by nosocomial infections, and he ultimately died.

This case illustrates an underrecognised thrombotic-predominant presentation of malaria-associated DIC, and highlights the diagnostic and therapeutic challenges associated with severe imported malaria in the intensive care setting.

## Introduction

Disseminated intravascular coagulation (DIC) is an acquired syndrome, secondary to an underlying acute illness, characterised by systemic activation of the coagulation cascade that extends beyond sites of vascular injury, resulting in diffuse intravascular fibrin formation, predominantly in small vessels [1]. It manifests as a thrombohaemorrhagic disorder in which widespread intravascular thrombosis occurs simultaneously with consumption of platelets and coagulation factors, potentially leading to microvascular obstruction, bleeding complications, and multiorgan dysfunction [2]. DIC is most frequently associated with severe infection and sepsis, followed by trauma, malignancy, immune and inflammatory diseases and obstetric complications, particularly in critically ill patients [3].

Malaria is a mosquito-borne parasitic infection caused by the *Plasmodium* species and remains a major global health problem, particularly in sub-Saharan Africa. Angola is among the countries with the highest malaria burden worldwide, while imported cases continue to be reported in non-endemic European settings. Among the species infecting humans, *Plasmodium falciparum* is associated with the highest morbidity and mortality due to its propensity to cause severe disease.

DIC is a recognised but relatively uncommon complication of malaria and occurs predominantly in severe forms of infection. The pathophysiology involves endothelial activation, parasite sequestration within the microvasculature, systemic inflammatory response, platelet activation, and dysregulation of coagulation pathways, leading to a prothrombotic state with potential secondary consumption of clotting factors. Disseminated intravascular coagulation (DIC) is primarily associated with severe *Plasmodium falciparum* malaria; however, recent evidence indicates a variable prevalence across species, with distinct rates reported for *P. falciparum* and* P. vivax* infections [4].

Classically, malaria-associated DIC has been described as a haemorrhagic phenotype; however, thrombotic-predominant presentations have been reported occasionally and remain incompletely characterised in the literature [5]. Severe *Plasmodium falciparum* malaria may precipitate multiple organ dysfunction, including acute renal failure, cerebral involvement, shock, and life-threatening coagulopathy requiring intensive care support [4].

In this report, we describe a rare presentation of severe *Plasmodium falciparum* malaria complicated by DIC with a predominant thrombotic phenotype, highlighting an underrecognised and underreported manifestation of this serious tropical infection and its implications in the intensive care setting.

## Case presentation

A 61-year-old man (weight 100 kg) presented to the emergency department (ED) with a 4-5-day history of progressive malaise, intermittent diaphoresis and subjective fever, with a documented temperature of 38.4°C at home. His medical history included type 2 diabetes mellitus, diagnosed approximately 10 years earlier and treated with oral hypoglycaemic agents, without known microvascular complications, and peripheral artery disease with intermittent claudication but no prior limb ischemia, revascularisation procedures or amputation. On the day of admission, he developed sudden-onset severe diffuse abdominal pain (rated 10/10), non-radiating, associated with repeated vomiting and increasing agitation. He had returned one to two weeks earlier from Angola, where he had stayed for three weeks, without having taken malaria chemoprophylaxis.

**Figure 1:**
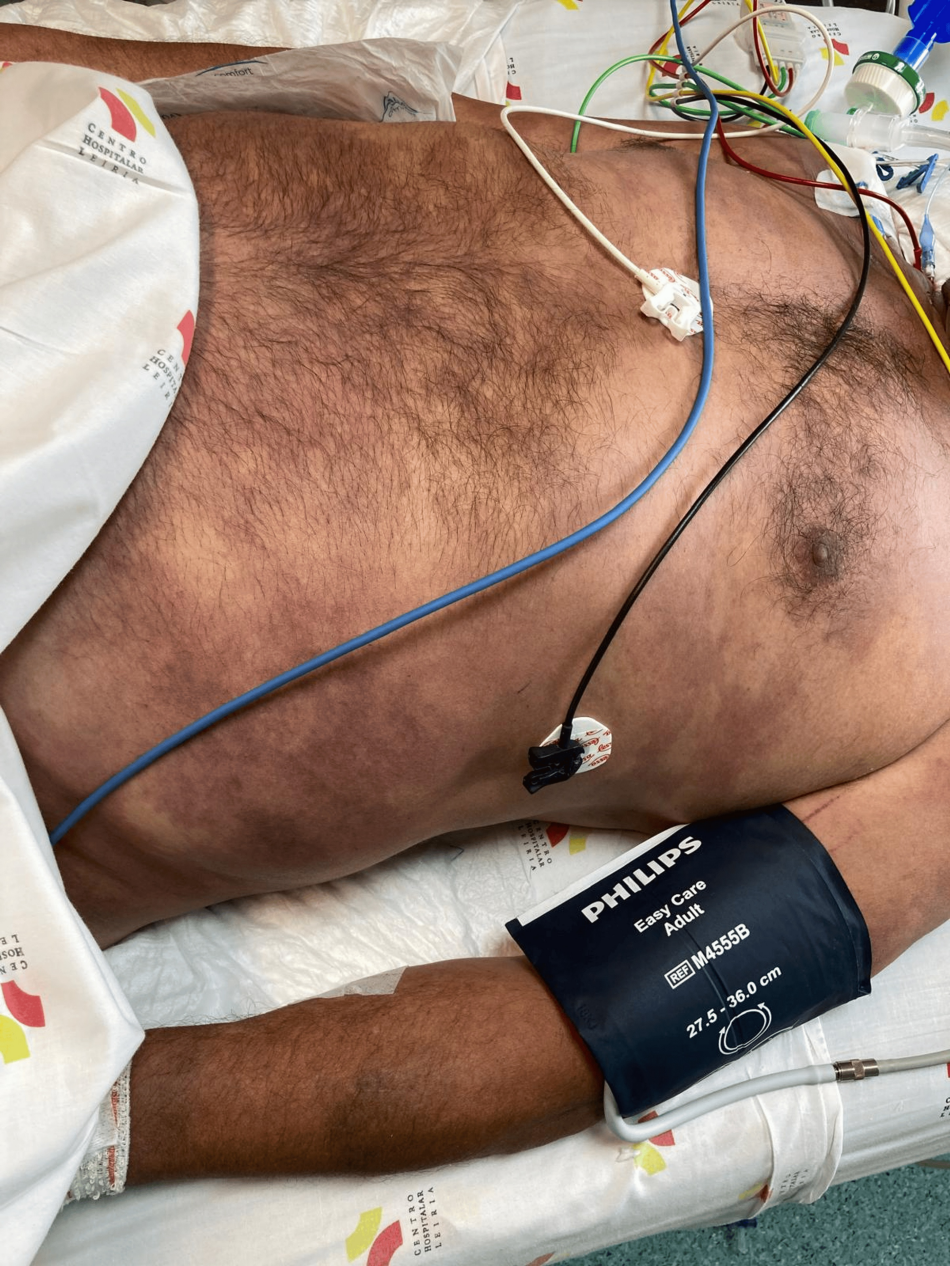
Cutaneous signs of severe hypoperfusion involving the trunk (diffuse dypatchy violaceous discoloration consistent with peripheral vasoconstriction and impaired microcirculatory perfusion) can be observed.

**Figure 2:**
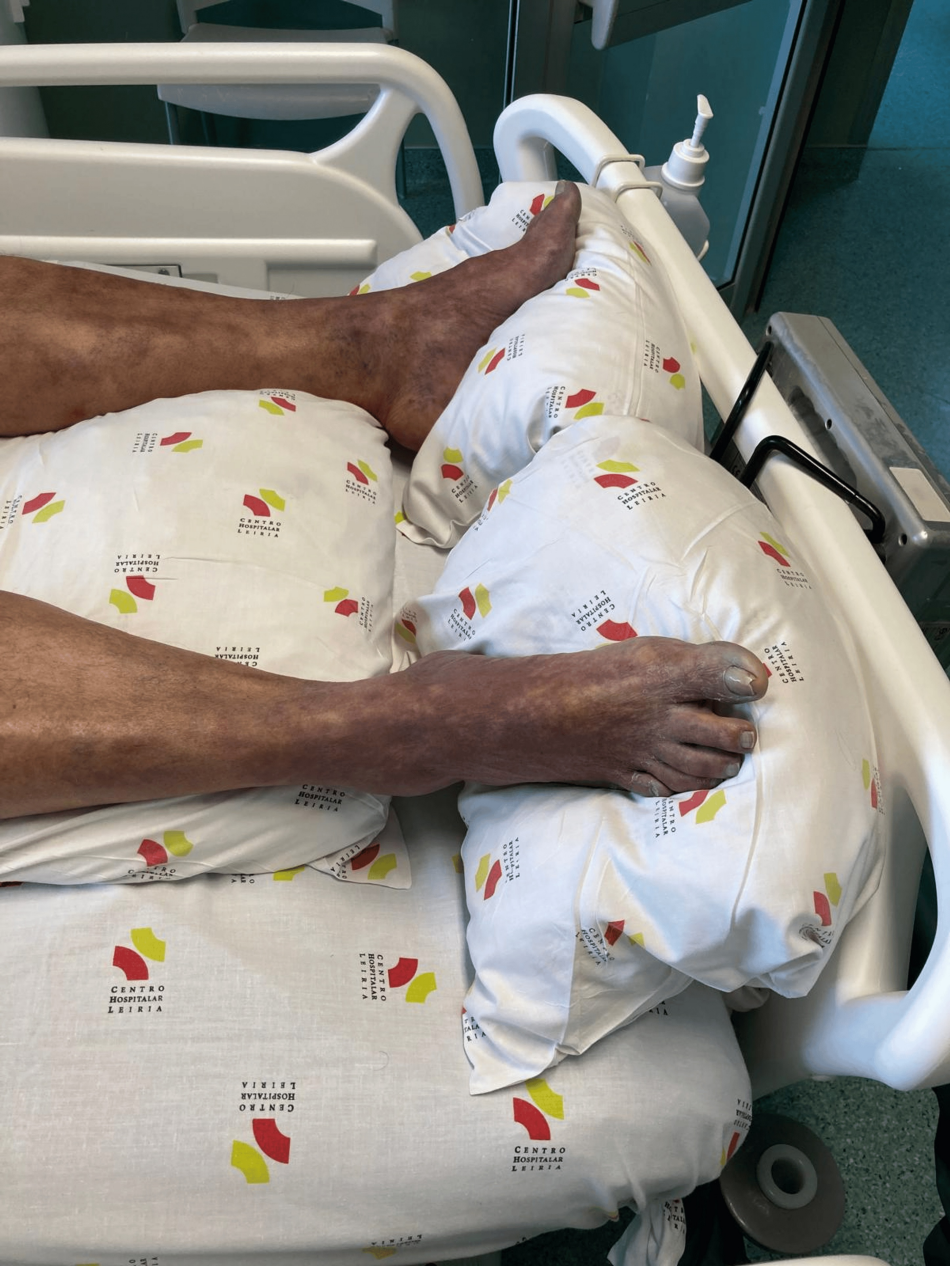
Marked peripheral cyanosis.

Physical examination revealed signs of severe hypoperfusion, including cold and mottled skin involving the trunk and all four limbs, marked peripheral cyanosis, profuse diaphoresis, tachypnea, agitation and confusion (Figures 1, 2). There were no signs of active bleeding. Peripheral oxygen saturation was 97% on room air, heart rate was 95 beats per minute, body temperature was 38.5°C, and capillary blood glucose was 110 mg/dL. Blood pressure measurements in all four limbs were asymmetric, with values of 194/106 mmHg in the left upper limb, 195/105 mmHg in the right upper limb, 202/105 mmHg in the left lower limb, and 111/90 mmHg in the right lower limb. 

According to previous medical records, his baseline serum creatinine was 0.9 mg/dL three months prior to admission. Initial laboratory evaluation demonstrated severe metabolic acidosis, hyperlactatemia, acute kidney injury, markedly elevated inflammatory markers, profound thrombocytopenia and coagulation abnormalities consistent with DIC (Table 1).

**Table 1:** Main laboratory results at the emergency department presentation. CrCl: creatinine clearance; pCO₂: partial pressure of carbon dioxide; INR: international normalized ratio; ALT: alanine aminotransferase; AST: aspartate aminotransferase; LDH: lactate dehydrogenase; aPTT: activated partial thromboplastin time.

Parameter	Result	Reference range	Clinical interpretation
Arterial blood gas			
pH	7.3	7.35–7.45	Metabolic acidosis
pCO₂ (mmHg)	18	35–45	Respiratory compensation
Bicarbonate (mmol/L)	8.9	22–26	Severe metabolic acidosis
Lactate (mmol/L)	11.1	<2.0	Severe hyperlactatemia
Renal function			
Creatinine (mg/dL)	2.06	0.7–1.3	Acute kidney injury
Urea (mg/dL)	91	10–50	Renal dysfunction
CrCl (mL/min)	53	>90	Moderate impairment
Inflammation			
C-reactive protein (mg/L)	251	<5	Markedly elevated
Procalcitonin (ng/mL)	92	<0.05	Severe systemic inflammation
Hematology			
Platelet count (/µL)	9,000	150,000–400,000	Profound thrombocytopenia
Leukocyte count (/µL)	4,300	4,000–10,000	Within normal range
Hemoglobin (g/dL)	15.9	13–17	Within normal range
Coagulation			
D-dimer (ng/mL)	>12,800	<500	Markedly elevated
INR	1.18	0.8–1.2	Mild prolongation
Prothrombin activity (%)	79	70–100	Near normal
aPTT (seconds)	41.1	25–35	Mild prolongation
Fibrinogen (mg/dL)	489	200–400	Preserved/normal-high
Liver / tissue injury			
Total bilirubin (mg/dL)	3.2	<1.2	Hyperbilirubinemia
ALT (U/L)	82	<40	Mild elevation
AST (U/L)	201	<40	Marked elevation
LDH (U/L)	827	<250	Tissue injury/hemolysis
Pancreatic enzymes			
Amylase (U/L)	125	25-125	Within normal range
Lipase (U/L)	110	13-60	Slightly elevated
Electrolytes			
Sodium (mmol/L)	137	136-146	Within normal range
Potassium (mmol/L)	3.7	3.5-5.1	Within normal range
Cardiac biomarkers			
Troponin (pg/mL)	42.4	<19.8	Slightly elevated

Given the combination of recent travel to Angola, a febrile episode, profound thrombocytopenia, severe hyperlactatemia, and acute kidney injury, severe malaria was considered early in the diagnostic evaluation. Although bacterial sepsis with DIC was initially included in the differential diagnosis, the absence of a clear bacterial source on initial clinical and imaging evaluation, the marked thrombocytopenia disproportionate to leukocyte count, and the epidemiological exposure raised strong suspicion for imported malaria. Empirical broad-spectrum antibiotic therapy with IV piperacillin-tazobactam 1(8g/day perfusion) was initiated on admission pending microbiological work-up, while urgent peripheral blood smear and rapid antigen testing were performed to evaluate for *Plasmodium* infection.

Peripheral blood smear demonstrated erythrocytes with anisopoikilocytosis, anisocytosis, and polychromasia, with frequent ring forms. Rapid antigen testing and microscopy confirmed *Plasmodium falciparum* infection, with an estimated parasitemia exceeding 25%.

Computed tomography of the chest, abdomen, and pelvis revealed diffuse atherosclerotic disease with partial thrombotic involvement of the distal aorta and mesenteric vessels, without evidence of acute large-vessel occlusion or surgical abdominal pathology. The patient rapidly progressed to distributive shock with evolving multiorgan dysfunction, requiring endotracheal intubation, invasive mechanical ventilation, vasopressor support and was admitted to the intensive care unit (ICU) with a diagnosis of severe *Plasmodium falciparum* malaria complicated by DIC with predominant thrombotic features and multiorgan dysfunction. 

Intravenous artesunate was promptly initiated at a dose of 240 mg (at 0, 12 and 24 hours, then daily) and continued for six days, followed by a three-day course of artemisinin-based combination therapy (artemether-lumefantrine). Given the thrombotic-predominant presentation of DIC and absence of active bleeding, anticoagulation with unfractionated heparin was initiated under close hematologic monitoring (continuous infusion at 10 U/kg/hour, without an initial bolus). The patient required multiorgan support, including vasopressor therapy (maximum norepinephrine dose of approximately 1 µg/kg/min on day 1), invasive mechanical ventilation, renal replacement therapy for oligoanuric acute kidney injury with persistent acidemia, and transfusional support with platelet concentrates (a total of four pooled platelet units).

Serial blood smears demonstrated a rapid and progressive reduction in parasitemia, decreasing to below 5% within four days of treatment initiation (day 1 >25%, day 2: 17.5%, day 3: 15%, day 4: <5%), followed by complete parasitological clearance. Despite parasitological control, early initiation of anticoagulation and hemodynamic stabilisation (vasopressor dose reduced by more than half on day 2 and discontinued by day 4), the patient developed progressive ischemia of the lower extremities, most pronounced in the right leg, evolving to tissue necrosis. A Doppler ultrasound of the lower limbs performed on day 5 revealed an obliterative process of the right femoropopliteal axis due to arterial thrombosis, with markedly reduced distal flow and no evidence of deep venous thrombosis. After multidisciplinary discussion, a right above-knee amputation was performed on day 11.

During the subsequent ICU course, the patient developed multiple nosocomial infections, including bacterial pneumonia and pulmonary aspergillosis, requiring targeted antimicrobial therapy with iv meropenem (1g 8/8h) and linezolid (600mg 12/12h) for bacterial pneumonia and antifungal treatment with iv caspofungin (50 mg q24h) followed by iv voriconazole (8mg/kg/day) for pulmonary aspergillosis. He was eventually discharged from the ICU following partial clinical recovery. Three weeks later, the patient was readmitted to the ICU with acute hypoxemic respiratory failure and decreased level of consciousness in the context of persistent pulmonary infection and progressive respiratory compromise. At admission, he required high-flow oxygen therapy due to worsening gas exchange. During his ICU course, he experienced a massive aspiration event leading to sudden clinical deterioration and cardiac arrest with pulseless electrical activity. Advanced life support was initiated promptly; however, sustained return of spontaneous circulation could not be achieved.

## Discussion

DIC is a complex acquired hematological syndrome characterised by widespread activation of the coagulation cascade, resulting in both microvascular thrombosis and, paradoxically, life-threatening bleeding [1,2]. It almost invariably arises as a secondary complication of an underlying disease. This condition is mostly caused by severe infection (sepsis), injury, cancer, or pregnancy complications, and less often by other types of infection [1]. Malaria represents one such trigger; however, despite its global disease burden, its association with overt DIC remains relatively uncommon but potentially devastating. The prevalence of DIC among malaria patients is estimated at approximately 11.6%, increasing to 14.6% in severe *Plasmodium falciparum* infection, and up to 82.2% in fatal cases [4]. Importantly, malaria-associated DIC has classically been described as hemorrhagic, whereas thrombotic-predominant presentations remain rare and substantially underreported [4,5]. The present case exemplifies this uncommon phenotype, highlighting the heterogeneity of DIC manifestations in severe malaria and the need for heightened clinical awareness of non-classical presentations.

The pathophysiology of thrombotic DIC in severe *Plasmodium falciparum* malaria reflects profound dysregulation of the hemostatic system, encompassing excessive coagulation activation, impairment of endogenous anticoagulant pathways, and suppression of fibrinolysis [2,4,5]. Intense inflammatory responses driven by a high parasite burden promote tissue factor expression on monocytes and activated endothelial cells, initiating and amplifying thrombin generation [4,5]. Concurrently, natural anticoagulants, such as antithrombin and the protein C and S systems, are depleted or functionally impaired through consumption, increased vascular permeability, hepatic dysfunction, and cytokine-mediated inhibition [2,4]. Endothelial activation further contributes by increased expression of adhesion molecules and the release of von Willebrand factor, thereby enhancing platelet adhesion and microvascular thrombosis [5]. A defining feature of thrombotic-predominant DIC is fibrinolytic shutdown, mediated by elevated plasminogen activator inhibitor-1 levels, which prevents fibrin degradation and promotes pathological fibrin accumulation within the microcirculation [2,4].

The downstream consequence of these processes is widespread microvascular thrombosis, leading to tissue ischemia and multiorgan dysfunction [2,4]. Pathological studies in severe malaria have demonstrated extensive fibrin deposition within the microvasculature, contributing to acute kidney injury, respiratory failure, hepatic dysfunction, and cerebral involvement [4]. Peripheral ischemia and arterial thrombosis represent particularly severe manifestations of this systemic prothrombotic state [2,6]. In the present case, progressive lower-limb ischemia culminating in femoropopliteal arterial thrombosis and tissue necrosis occurred despite early anticoagulation and parasitological control, illustrating the fulminant and refractory nature that thrombotic DIC may assume. The marked mottling and asymmetric blood pressure measurements observed at presentation likely reflected early involvement of larger vascular territories, consistent with a diffuse thrombotic process affecting multiple vascular beds.

Comparison with published case reports further underscores the rarity of this presentation. Reported cases of malaria-associated DIC, including those involving *Plasmodium vivax* and *Plasmodium falciparum*, typically describe profound thrombocytopenia, elevated D-dimer levels, and multiorgan dysfunction, but more often emphasise hemorrhagic manifestations rather than arterial thrombosis [7-9]. In contrast, the current case was characterised by predominant arterial thrombosis leading to peripheral ischemia and limb loss despite appropriate antimalarial therapy and anticoagulation. This divergence suggests that thrombotic-predominant DIC may represent a distinct pathophysiological trajectory, potentially driven by heightened endothelial activation in the setting of extreme parasitemia exceeding 25%. Peripheral arterial ischemia and thrombosis, therefore, emerge as markers of particularly severe endothelial dysfunction and thromboinflammation. While most published cases of malaria-associated DIC focus on hemorrhagic or multiorgan involvement, documented arterial thrombosis resulting in limb-threatening ischemia remains exceptionally uncommon [6].

Diagnosing thrombotic DIC in critically ill patients remains challenging, as coagulation abnormalities frequently overlap with those observed in sepsis and systemic inflammatory states [2]. Profound thrombocytopenia, markedly elevated D-dimer levels, mildly prolonged coagulation times, and preserved or normal fibrinogen levels, as observed in this patient, are characteristic of thrombotic DIC, in which active fibrin formation and impaired fibrinolysis favor thrombosis over bleeding [2,10]. Recognition of the underlying malarial etiology is therefore essential, as early parasitological diagnosis through blood smear microscopy and antigen testing allows timely distinction from other causes of critical illness-associated coagulopathy and enables prompt etiological treatment.

Management of thrombotic DIC in severe *Plasmodium falciparum* malaria rests on two interdependent principles: rapid eradication of the parasite and meticulous supportive care [2,3]. Intravenous artesunate remains the treatment of choice for severe malaria, achieving rapid parasite clearance and reduction of the inflammatory and procoagulant stimulus [3,7]. Supportive management requires careful transfusion strategies to address consumptive coagulopathy while avoiding exacerbation of hypercoagulability. The role of anticoagulation remains controversial and must be individualised. While unfractionated heparin may theoretically mitigate microvascular thrombosis, current evidence emphasises treatment of the underlying cause as the primary intervention, with anticoagulation reserved for selected cases with predominant thrombotic manifestations [2,3]. The present case demonstrates that despite aggressive etiological treatment, supportive transfusion, hemodynamic optimisation, and anticoagulation, irreversible microvascular and macrovascular thrombosis may still occur, underscoring the limitations of current therapeutic approaches and highlighting the need for further investigation into optimal management strategies for thrombotic-predominant DIC in malaria.

## Conclusions

DIC represents a rare but severe complication of malaria, particularly in cases of severe *Plasmodium falciparum* infection. Malaria triggers a complex inflammatory cascade characterised by endothelial activation, cytokine release, and coagulation pathway dysregulation, which may precipitate DIC in the most severe presentations. While malaria-associated DIC is classically described as haemorrhagic, this case highlights an uncommon thrombotic-predominant phenotype characterised by extensive microvascular and macrovascular thrombosis with devastating clinical consequences. Early recognition of non-classical DIC phenotypes, prompt parasitological diagnosis, and rapid initiation of etiological treatment are essential but may not prevent thrombotic complications in the most severe presentations. This case underscores the heterogeneity of DIC manifestations in malaria and highlights the need for increased clinical awareness and further research into optimal management strategies for thrombotic-predominant DIC in this setting.

## References

[1] Iba T, Helms J, Connors JM, Levy JH (2023). The pathophysiology, diagnosis, and management of sepsis-associated disseminated intravascular coagulation. J Intensive Care.

[2] Gando S, Levi M, Toh CH (2025). Disseminated intravascular coagulation. J Intensive Care.

[3] Levi M, Scully M (2018). How I treat disseminated intravascular coagulation. Blood.

[4] Duangchan T, Kotepui M, Sukati S, Rattanapan Y, Wangdi K (2023). A systematic review and meta-analysis of the proportion estimates of disseminated intravascular coagulation (DIC) in malaria. Trop Med Infect Dis.

[5] O'Sullivan JM, Preston RJ, O'Regan N, O'Donnell JS (2016). Emerging roles for hemostatic dysfunction in malaria pathogenesis. Blood.

[6] Gong F, Zheng X, Zhao S (2025). Disseminated intravascular coagulation: cause, molecular mechanism, diagnosis, and therapy. MedComm (2020).

[7] Lippmann A, Samavati S, Awan M (2025). A case of severe malaria with disseminated intravascular coagulation in an American missionary and review of current treatment and prophylaxis. Clin Infect Immun.

[8] Sailo L, Pradhan D, Nongthombam R, Bhattacharyya P (2014). Disseminated intravascular coagulation in malaria: a case report. Niger Med J.

[9] Satti Z, Khurshid A, Mohammed R, Jose R, Olayode A (2023). Plasmodium vivax presenting with septic shock and disseminated intravascular coagulation (DIC): a case report. Cureus.

[10] Iba T, Levy JH, Maier CL (2025). Updated definition and scoring of disseminated intravascular coagulation in 2025: communication from the ISTH SSC Subcommittee on Disseminated Intravascular Coagulation. J Thromb Haemost.

